# *neurotic*: Neuroscience Tool for Interactive Characterization

**DOI:** 10.1523/ENEURO.0085-20.2020

**Published:** 2020-05-11

**Authors:** Jeffrey P. Gill, Samuel Garcia, Lena H. Ting, Mengnan Wu, Hillel J. Chiel

**Affiliations:** 1 Department of Biology, Case Western Reserve University, Cleveland, OH 44106-7080; 2 Centre de Recherche en Neuroscience de Lyon, Centre National de la Recherche Scientifique Unité Mixte de Recherche 5292– Institut National de la Santé et de la Recherche Médicale U1028–Université Claude Bernard Lyon 1, Lyon, 69675 Bron CEDEX, France; 3 The Wallace H. Coulter Department of Biomedical Engineering, Emory University and Georgia Tech, Department of Rehabilitation Medicine, Division of Physical Therapy, Emory University School of Medicine, Atlanta, GA 30322-1119; 4 The Wallace H. Coulter Department of Biomedical Engineering, Emory University and Georgia Tech, Atlanta, GA 30322-1119; 5 Departments of Biology, Neurosciences, and Biomedical Engineering, Case Western Reserve University, Cleveland, OH 44106-7080

**Keywords:** behavioral neuroscience, data sharing, open source, Python, video synchronization, visualization

## Abstract

A software tool for synchronization of video with signals would be of broad general use to behavioral neuroscientists. A new program, called *neurotic* (NEUROscience Tool for Interactive Characterization), allows users to review and annotate signal data synchronized with video, performs simple initial analyses including signal filtering and spike detection, is easy to use, and supports a variety of file formats. The program also facilitates collaborations by using a portable specification for loading and processing data and retrieving data files from online sources. Two examples are shown in which the software is used to explore experimental datasets with extracellular nerve or muscle recordings and simultaneous video of behavior. The configuration specification for controlling how data are located, loaded, processed, and plotted is also summarized. Algorithms for spike detection and burst detection are demonstrated. This new program could be used in many applications in which behavior and signals need to be analyzed together.

## Significance Statement

Behavioral neuroscience would benefit from a tool that allows easy visualization of both behavior and neural or other signals. A new tool, called *neurotic* (NEUROscience Tool for Interactive Characterization), is described that is free and open-source and can make data visualization, analysis, and collaboration much easier.

## Introduction

In behavioral neuroscience, to interpret data after it is collected, it is essential that behavioral video be synchronized with electrophysiology. Although some commercial data acquisition systems may provide this functionality using proprietary hardware, software, and data formats [e.g., Plexon CinePlex Software (Jacobson et al., 2014), Blackrock Microsystems NeuroMotive], and although specialized tools exist for separately analyzing signals or behavioral videos using licensed analysis programs like MATLAB (http://www.mathworks.com/solutions/neuroscience), there are few free and open-source software tools with the capability to synchronize video and neural signals. Although there are many open source neuroscience tools and libraries for analysis of neural signals alone [e.g., Open Ephys (http://open-ephys.org) and NeuralEnsemble (http://neuralensemble.org) are communities focused on developing open source tools for neuroscientists], they often have steep learning curves and require that users program their own scripts. Especially appealing would be an open source application that facilitated the review of electrophysiology data with synchronized video, was simple to install on any computer, easy for non-experts to use, and powerful enough for experienced data scientists to use in their data pipelines.

We have developed *neurotic* (NEUROscience Tool for Interactive Characterization), a Python tool with these capabilities (for important links, see Table 1). A graphical user interface (GUI) provides interactive plots of raw electrophysiology data, simultaneous video, and simple annotation tools. The program also performs basic data processing, like filtering and spike detection. Since it is built on the Neo library (Garcia et al., 2014), it can read many common electrophysiology file formats. The application is easily installed using common Python tools (pip or conda) or standalone installers, with built-in examples running quickly. After creating human-readable configuration files, users can explore data with the GUI. Portable configuration files and a data-fetching feature facilitate collaboration. Programmers can bypass the GUI and use the application programming interface (API) in their analysis pipelines in scripts and Jupyter notebooks (Kluyver et al., 2016).

**Table 1:** Important links

Resource	URL
Source code	https://github.com/jpgill86/neurotic
Documentation	https://neurotic.readthedocs.io
PyPI distribution	https://pypi.org/project/neurotic
Anaconda distribution	https://anaconda.org/conda-forge/neurotic
Standalone installers	https://github.com/jpgill86/neurotic/releases
Issue tracker	https://github.com/jpgill86/neurotic/issues

## Materials and Methods

### Software overview

*neurotic* relies on the Python library Neo (Garcia et al., 2014) for reading electrophysiology files, including formats like Axon, AxoGraph, Blackrock, Intan, and Open Ephys files; it also includes some generic formats like MATLAB MAT-files, HDF5, plain text ASCII, and generic binary data files, provided they are structured properly. Neo manages units (e.g., ensuring signals measured in millivolts and microvolts are summed correctly) using the quantities package (http://pypi.org/project/quantities), and both rely on the ubiquitous Numpy library (van der Walt et al., 2011). *neurotic* can take advantage of a feature of many of Neo’s file readers, which is to read slices of large files as needed, rather than loading entire files into memory at once; this “fast loading” feature greatly reduces load times and memory consumption.

*neurotic* reads spreadsheet files (CSV format) using the Python library pandas (McKinney, 2010) to import epochs and events. Epochs are labeled periods of time with a beginning and an end which are used to annotate data by labeling periods of interest. Events are labeled points in time that serve as “bookmarks” into the experiment, allowing users to jump to them. The *neurotic* interface allows users to create and save epochs to a spreadsheet.

*neurotic* can read many video file formats using the PyAV library (http://pypi.org/project/av). Sound stored in video files is currently ignored by *neurotic*.

*neurotic* can perform processing and analysis procedures on the data, including applying filters, generating spike trains from signal peaks, and identifying bursts (periods of high activity in the spike trains). These procedures are aided by the Python library elephant (http://pypi.org/project/elephant), which provides advanced algorithms for analyzing Neo objects. Many of these algorithms are applications of signal processing procedures implemented in the SciPy package (Virtanen et al., 2020).

**Figure 1.**
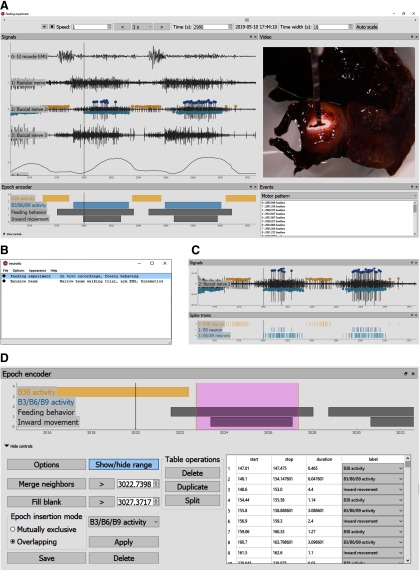
GUI. The GUI of *neurotic* combines data from multiple sources, such as electrophysiology data files and behavioral video files, into a unified and synchronized view for reviewing and annotating experimental data. This example shows data collected as an animal (*Aplysia californica*) ate seaweed (see Materials and Methods, Experimental methods). Implanted electrodes captured extracellular signals from a muscle and three nerves associated with feeding. Video of behavior and swallowing force from food attached to a force transducer were also recorded. ***A***, The interface has movable and resizable panels created using the Python package ephyviewer (http://pypi.org/project/ephyviewer). Across the top of the interface, a time navigation bar allows a user to jump through the record or play it at different speeds (keyboard shortcuts provide similar features). The “time width” box controls the displayed duration. Muscle, nerve, and force signals are displayed (top left panel) along with the video (top right panel). A vertical line indicates the time corresponding to the displayed video frame. Panels can be zoomed using the mouse, and time can be expanded or contracted using the mouse or the time width input. User-defined events are listed (bottom right panel), providing bookmarks to different parts of the experiment. A tool called the epoch encoder (bottom left panel) allows users to label time periods (controls are collapsed here; see ***D*** for details). Some panels can be double-clicked to open dialogue windows with additional options (e.g., to hide plotted signals). ***B***, Before displaying a user interface like that in ***A***, *neurotic* provides a window for selecting datasets. Users load a dataset list from a manually created human-readable YAML configuration file that provides paths to data files and other parameters (Fig. 2). If data and video files are available on the internet, *neurotic* can be configured to locate and download files if they are not already available locally. Icons to the left of each dataset name indicate whether files are already available locally or not. This window provides menus for loading options and color themes. The most important of these is the toggleable fast loading option, which is available for a subset of data file types and provides much faster loading with less memory consumption in exchange for skipping data processing steps like filtering and spike detection. ***C***, Using optional parameters provided in the configuration file (Fig. 2*G*), peaks can be detected on signals using amplitude-threshold window discriminators (see Fig. 3 for algorithm). Peaks identified by the same discriminator are grouped as a unit and plotted both as points on the signals and as raster plots in a separate panel (hidden in ***A*** but shown in this panel). ***D***, The epoch encoder panel displays horizontal bars to represent user-created, labeled time periods. Periods of interest, such as a particular behavior, the phase of a rhythmic behavior, or the activity of an identified neuron may be labeled using the epoch encoder. These data are saved to a plain text spreadsheet format (CSV) that may be used for further analysis. The epoch encoder panel allows precise control of the temporal parameters of epochs, merging of adjacent epochs, and filling gaps between epochs. Epochs can be placed using the range selection feature (magenta rectangle) with the mouse by dragging its edges. Keyboard shortcuts exist for rapid encoding. A table lists the existing epochs and allows them to be modified or removed.

Finally, *neurotic* uses the Python library ephyviewer (http://pypi.org/project/ephyviewer) for plotting and interacting with signals, epochs, events, spike trains, and video frames. ephyviewer is built on the pyqtgraph library (http://www.pyqtgraph.org), which creates a Qt application interface (www.qt.io). Figure 1*A* shows the *neurotic* user interface for an example dataset in which neural and force data were collected as an animal fed (Gill and Chiel, 2020). Electrophysiology signals are plotted after filtering, and points mark peaks identified by window discriminators. Video shows the animal’s behavior, and the timing of the displayed frame is indicated by a vertical line superimposed on the plots. A navigation panel allows the user to simultaneously move through the neural data and synchronized video. Another panel (“epoch encoder”) displays user-created epochs as horizontal bars and provides controls for creating and modifying epochs (collapsed in Fig. 1*A*; for details, see Fig. 1*D*). A list of user-designated events makes it possible to jump to moments of interest.

### Software configuration

*neurotic* requires configuration before use with a dataset. For example, the paths to the electrophysiology data file and behavioral video file must be specified. Configurations are saved in the human-readable YAML file format which can be used to load and process data repeatedly.

**Figure 2.**
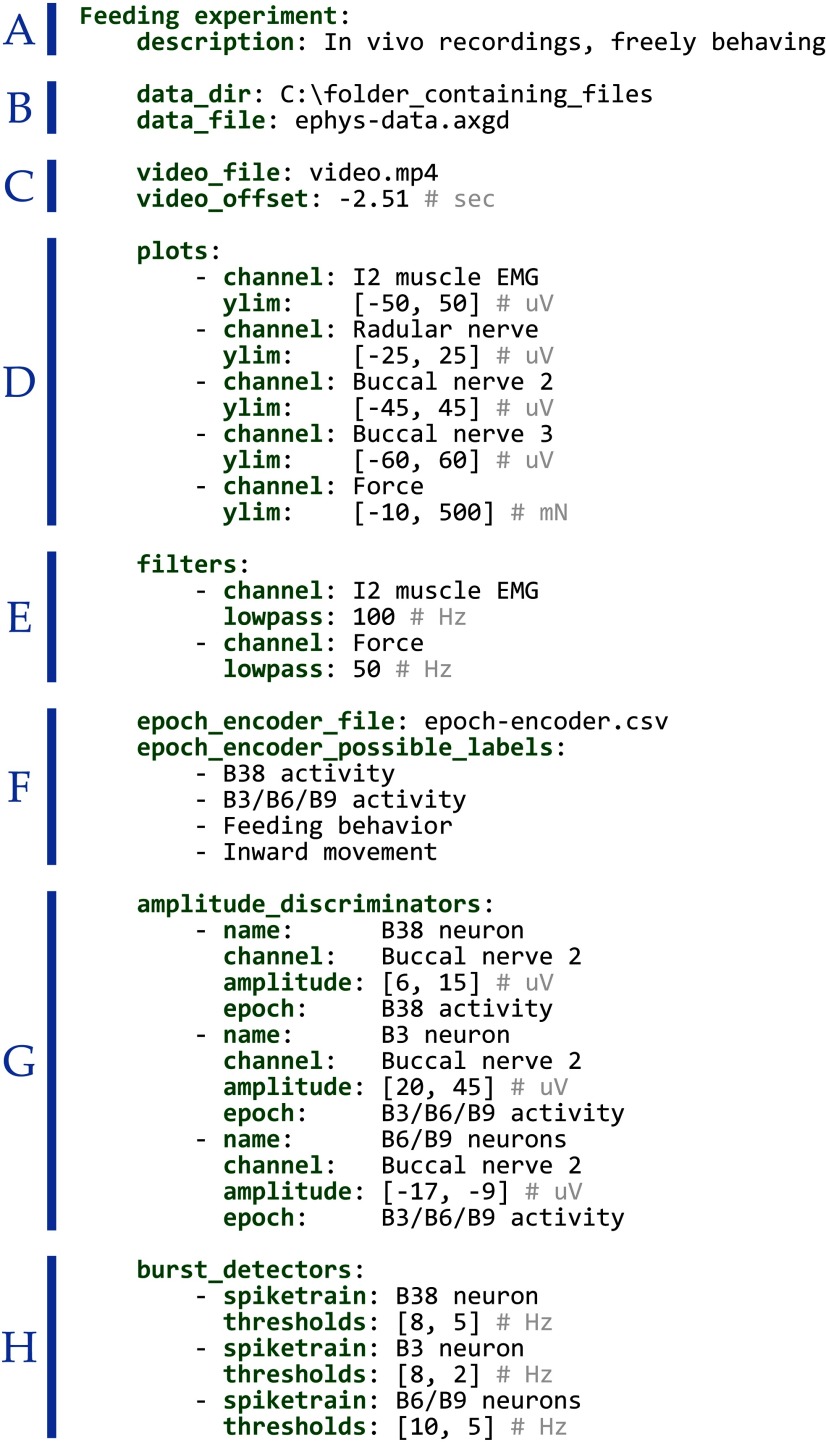
An example configuration file. To visualize experimental data with *neurotic*, datasets must be listed and described within human-readable plain text YAML configuration files. Multiple configurations for the same or different datasets may be listed within one file and are separated into blocks using blank lines and indentation. This example shows one configuration that is similar to that used to create Figures 1, 3. Each set of parameters shown is optional. ***A***, Each configuration is given a unique name, e.g., “feeding experiment.” Details associated with the configuration are indented beneath the name. A brief description of the dataset may be provided. The names and descriptions of datasets are displayed when a YAML file is loaded, giving the user the option to select the dataset to view (Fig. 1*B*). ***B***, Paths to related files found locally on the computer, e.g., signals and video from a single experiment, may be located within a single directory specified by data_dir (such a file tree structure is convenient but not necessary as *neurotic* can accept relative or absolute paths). A file containing signal data that is readable by the Python package Neo (Garcia et al., 2014) is specified using data_file. Neo supports many file types (see list: https://neo.readthedocs.io/en/latest/io.html#module-neo.io). Signals are read from the data file, processed according to other, optional configuration settings, and plotted. For example, in this figure, sections ***D***, ***E***, ***G*** affect plotting. ***C***, A video file may be associated with the signal data. Synchronization parameters can be given for controlling the video and signal data alignment. For example, in this case, video capture began 2.51 s before signal data acquisition, so −2.51 is provided for video_offset to shift the video start time. Parameters for correcting for frame rate inaccuracies or clipping the video are also available. ***D***, All signals or a subset of signals will be plotted according to the parameters given under plots, which control plot range and labeling (Fig. 1*A*, top left panel). ***E***, Signals may be filtered before plotting. ***F***, An optional epoch encoder GUI panel (Fig. 1*D*) creates annotations that may be saved to a spreadsheet (CSV) file. The epoch encoder allows user-defined labels to be attached to time periods. ***G***, Peaks may be identified in the signals using amplitude thresholds. Each amplitude discriminator is applied to the specified signal channel and may be constrained to periods marked with a particular epoch label. This creates one spike train for each amplitude discriminator, plotted both as points on the signals and as a raster plot (Fig. 1*C*). See Figure 3 for algorithm details. ***H***, Bursts of activity in spike trains may be detected using initiation and termination firing frequencies.

Figure 2 shows an example of a detailed configuration written in the YAML format. A minimal configuration could be much simpler, including, for instance, a single file path. The example is divided into eight sections: (1) a unique dataset name and brief description; (2) file paths may be listed relative to a primary directory; (3) video synchronization parameters; (4) signal plotting parameters; (5) signal filtering; and settings for (6) the epoch encoder, (7) peak detection, and (8) burst detection.

Multiple configurations like that shown in Figure 2 may be contained in a single YAML file. This allows multiple datasets, or alternate processing and display configurations for the same dataset, to be contained within one file. When *neurotic* is first opened and the configuration file is selected, a list of dataset configurations is displayed (Fig. 1*B*). The user can choose which configuration to load and can adjust options for how data are loaded and displayed.

For downloadable datasets, including those on private password-protected servers or public data repositories like GIN (http://gin.g-node.org), the configuration may include URLs for the files, and *neurotic* can download them. In combination with relative file paths, this makes configuration files fully portable, such that users may share them with collaborators and load datasets from other computers.

Synchronizing a video file and a signal data file captured using independent data acquisition systems may be complicated, since files may start at different times or the video may contain multiple trials. *neurotic* provides several options for synchronization, including options for offsetting the video start time, adjusting frame rate, and skipping sections.

### Algorithms for data processing

**Figure 3.**
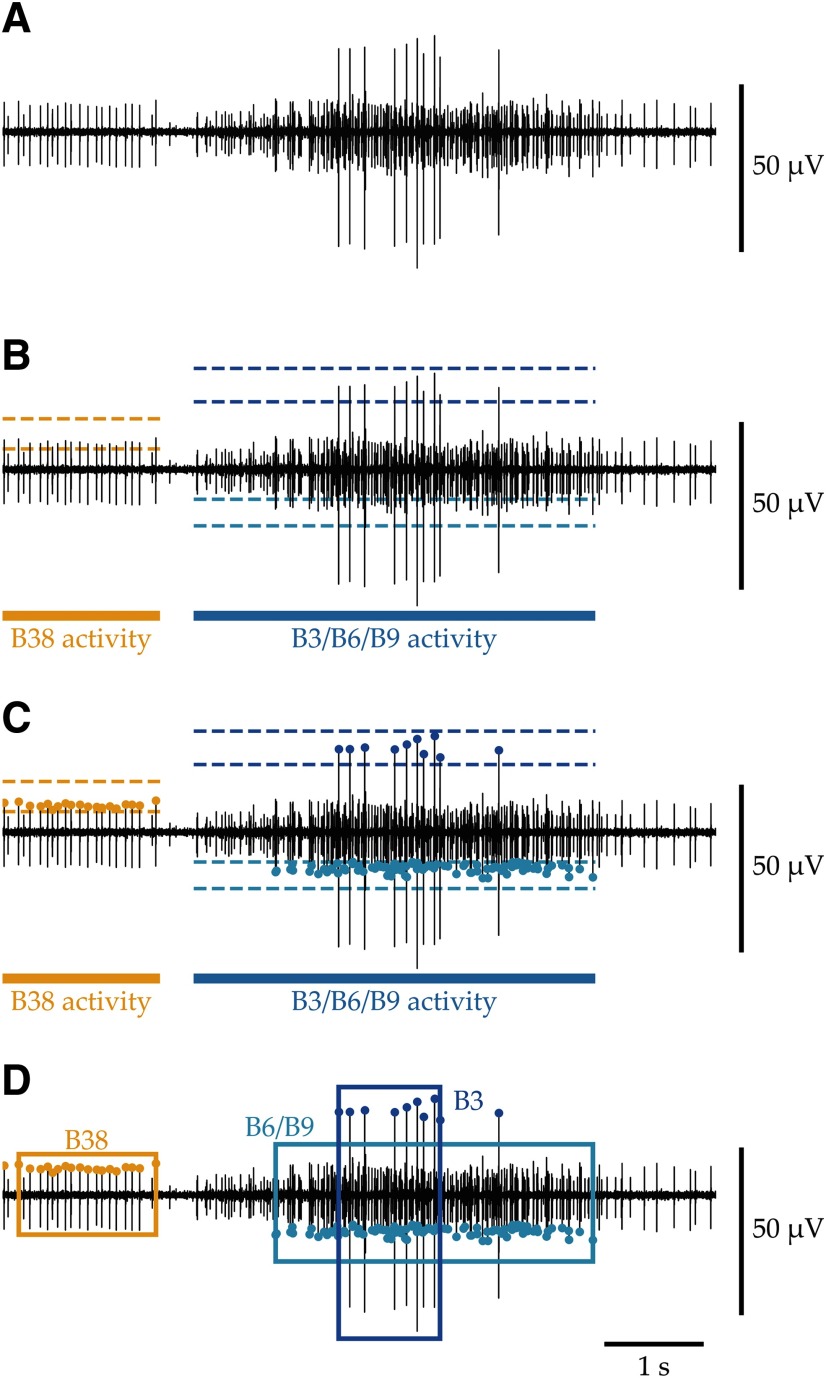
Peak detection using a window discriminator to define neuronal bursts. Panels show an example of applying three amplitude discriminators to a single channel to obtain three sets of peaks, classified as action potentials originating from identified motor neurons B38 (McManus et al., 2014), B6/B9, and B3 (Lu et al., 2015). The configuration settings are shown in Figure 2G. ***A***, The raw signal obtained from an extracellular whole nerve recording of BN2 is plotted and contains activity from motor and sensory neurons. Identified neurons are distinguishable by their relative amplitude and timing within the feeding motor pattern (Lu et al., 2013). ***B***, Epochs labeled “B38 activity” and “B3/B6/B9 activity” were placed manually using the epoch encoder (Fig. 1*D*) based on prior work. Amplitude windows for spike classes were estimated manually and specified in the configuration file. ***C***, At load time, using algorithms from the Python package elephant (http://pypi.org/project/elephant), local maxima or minima were located within the given amplitude range and contained within a period with an appropriate epoch label. These points are displayed on the signals and as raster plots (Fig. 1*C*). ***D***, Subsets of each spike train were grouped into bursts defined by firing frequency thresholds (boxes). Notice that the initial B38 spike and the final B3 spike were excluded from the bursts because they did not meet the firing frequency conditions. Burst times may then be used for further analysis.

A simple algorithm for detecting peaks is illustrated schematically in Figure 3. Figure 3*A* plots nerve activity measured extracellularly (see below, Experimental methods). Each spike is an action potential, and the spike size is determined by the axon size, its proximity to the electrode, and its complement of ion channels.

Prior work in *Aplysia* has shown that the amplitude and timing of distinct units can identify neurons (Morton and Chiel, 1993; Hurwitz et al., 1996; Lu et al., 2013, 2015; McManus et al., 2014; Cullins et al., 2015). Figure 3*B* shows amplitude thresholds and epochs that together define windows for peak detection. Each amplitude discriminator has lower and upper thresholds, and may be constrained to labeled epochs. Identified peaks (Fig. 3*C*) are plotted in the *neurotic* interface as points and separately as a spike train raster plot (Fig. 1*C*).

High-frequency activity (bursts), defined by initiation and termination frequency thresholds, can be identified after peak detection. The result of applying burst detectors (configured in Fig. 2*H*) to the example spike trains is shown by boxes enclosing bursts (Fig. 3*D*). In the *neurotic* interface, the timing of detected bursts is plotted as epochs.

*neurotic* can also calculate the rectified area under the curve (RAUC). This procedure optionally removes a baseline (mean or median) from a signal, performs full-wave rectification (absolute value), and then integrates the signal in bins of fixed duration. The integration step introduces smoothing (dependent on the bin size). The resulting time series is plotted in the *neurotic* interface. This helps visualize overall signal activity [e.g., electromyogram (EMG), which is known as integrated EMG (iEMG)].

### API for additional analyses, customization, and prototyping

*neurotic* functions as a standalone application and also as a library with classes and functions that may be used in scripts or Jupyter notebooks. With *neurotic’s* API, users can read configuration files, load data, and apply processing steps as in the GUI. The resulting data objects are represented using Neo’s object model (Garcia et al., 2014) and may then be used for additional analyses. For example, after identifying peaks, creating spike trains, and detecting bursts using a *neurotic* configuration stored in a YAML file or in memory as a Python dictionary, a user might statistically analyze burst durations, plot inter-spike interval histograms, create publication-quality figures using a plotting package, or export data as part of a larger analysis.

The *neurotic* package includes an example Jupyter notebook containing a tutorial which can serve as a starting point for new users, and documentation is provided for the API.

### Experimental Methods

#### Animal feeding experiments

Briefly, the methods for obtaining neural correlates of responses to changing load during feeding in intact animals are described (for details, see Gill and Chiel, 2020).

Multichannel differential hook electrodes made from fine stainless steel wires (25-μm diameter, stainless steel 316, heavy polyimide insulated, California Fine Wire) were implanted in adult *Aplysia californica* (South Coast Bio-Marine; Cullins and Chiel, 2010). Animals were first anesthetized with an injection of isotonic (333 mm) magnesium chloride and immersed in an ice bath. An incision was made in the side of the head, and hook electrodes were attached using Kwik-Sil biocompatible silicone adhesive (World Precision Instruments) to nerves and muscles of the animal’s feeding apparatus: the I2 protractor muscle, the radular nerve (RN), and buccal nerve 2 (BN2) and buccal nerve 3 (BN3). The incision was then sutured closed, and animals were allowed to recover for 1–3 d.

Animals were presented with food in the form of seaweed strips (Deluxe Sushi-Nori, nagai roasted seaweed, Nagai Nori USA INC). While animals fed, signals from the hook electrodes were amplified and filtered (Model 1700 Differential AC Amplifiers, A-M. Systems), digitized (NI PCIe-6251 and NI BNC-2111, National Instruments), and recorded at 5000 Hz using AxoGraph X (AxoGraph Scientific) in continuous acquisition mode and saved in the AxoGraph file format, which is readable by Neo. In some trials, double-sided tape was placed between two seaweed strips to create an unbreakable strip, and this was attached to a force transducer (model FT03E, Grass Instrument Division, Astro-Med Inc) oriented over the animal to measure downward pulling force during swallowing. Force was also amplified, low-pass filtered, digitized by a strain gauge conditioner (model 3170, Daytronic), and recorded using AxoGraph X.

Simultaneously, video of the behavior was captured at 30 frames per second using a webcam (Logitech HD Pro Webcam C920) and Logitech Webcam Software. Video was saved as MP4 files with H.264 video encoding. A digital counter (C342-0562 totalizing counter, Veeder-Root) was programmed to increment at 10 Hz when the recording software was started; the counter was visible in the video recording at the start and end of the experiment, and pulses to the counter were recorded in AxoGraph X. This allowed the difference in timing between the start of video acquisition and the subsequent start of signal acquisition to be determined after the experiment, ensuring that the video and signal data could be synchronized by *neurotic*.

#### Human beam walking experiments

The methods for assessing human balance performance and control strategies while walking on a narrow beam are described. The participant consented to the protocol approved by the Georgia Tech and Emory Institutional Review Boards.

The participant attempted to walk as far as possible across a narrow beam with left arm free and right arm held across the chest. The participant was instructed to stop if she uncrossed her right arm or stepped off the beam. No explicit instructions were given regarding stepping pattern or walking speed.

Video data, EMG signals from arm muscles, and kinematics of the body were recorded using a ten-camera motion capture system (Vicon). EMGs were obtained at 1200 Hz from surface electrodes placed over the following arm muscles: biceps, triceps, and wrist flexors and extensors. The 3-D locations of retro-reflective markers placed at the center of the clavicles and both heels were collected at 120 Hz, along with time-synched video data (Vicon Bonita) collected at 40 Hz in AVI format and later converted to MP4 files.

## Results

Using *neurotic* for reviewing behavioral video and synchronizing it with neural signals greatly reduces analysis time. Previously, alignment of video and neural signals, which were recorded and reviewed in separate programs with independent time bases, required that times of interest for every comparison had to be located separately in each program.

*neurotic* solves the data stream alignment problem for old and new datasets. Moving through the video and finding the corresponding signal is done automatically in a unified interface, allowing for continuous playback and skipping to different parts of the experiment, making review easier, faster, and less prone to error.

**Figure 4.**
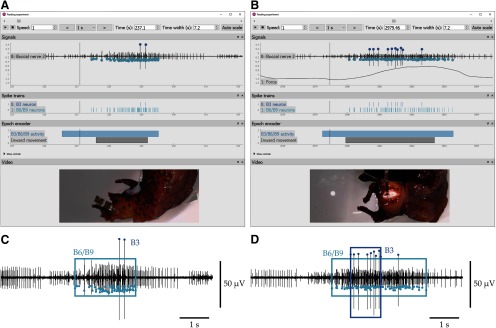
Using *neurotic* to explore an adaptive response to load during feeding. ***A***, As an animal swallowed a strip of unloaded (unanchored) seaweed, activity of identified feeding motor neurons B6/B9 and B3 was identified from the extracellular nerve recording of BN2. Using the synchronized video and epoch encoder, the timing of inward movement of the strip and the approximate period during which the identified neurons fired were labeled (gray and blue bars, respectively). Using the peak detection feature, medium-sized spikes were classified as B6/B9, and large spikes were classified as B3, which are major motor neurons known to contribute substantially to swallowing force (Lu et al., 2015). Spikes are plotted as points and in raster plots. ***B***, In a separate trial, the same animal fed on a strip of unbreakable seaweed anchored to a force transducer. The force generated by the animal is plotted with the extracellular nerve signals. In response to the increased load, the animal activated the identified motor neurons at high frequency for longer, leading to greater force. ***C***, Using firing frequency criteria determined by prior work (Lu et al., 2015), all spikes generated when feeding on an unloaded seaweed strip associated with the B6/B9 motor neurons (light blue) were grouped into a burst. The two spikes generated by B3 (dark blue) did not meet the criterion. ***D***, Using the same criteria when the animal fed on a loaded strip, all B6/B9 spikes and all but one B3 spike were grouped into bursts. The data suggest that the B3 neuron is more likely to activate at high frequency when the animal encounters load, and the B6/B9 neurons are active for a longer duration (Gill and Chiel, 2020).

To illustrate the program’s utility, we show alignment of behavior video with neural signals and force records (Fig. 4). Using *neurotic*, initial analysis of effects of mechanical load on feeding in *Aplysia* was simplified. Swallow durations were greater in the presence of load, and motor pattern changes could be quickly surveyed during multiple responses. For example, one swallowing response under conditions of low load (Fig. 4*A*) or high load (Fig. 4*B*) could be easily compared; only one neural channel (BN2) is plotted for clarity, as is force of swallowing when the seaweed strip was attached to the force transducer (Fig. 4*B*). Using the synchronized video, the periods during which the animal pulled food into its mouth could be identified, and these periods could be labeled using the epoch encoder (gray bars). Using amplitude window discriminators, action potentials generated by identified motor neurons could be located and represented as spike trains (raster plots). Using user-specified firing rate thresholds, *neurotic* detected bursts in the spike trains (Figs. 4*C*,*D*), allowing the durations of bursts to be compared when load was low or high. In the example, the duration of intense B6/B9 motor neuronal activity is longer during swallowing on a high-load stimulus, and the B3 motor neuron is recruited.

**Figure 5.**
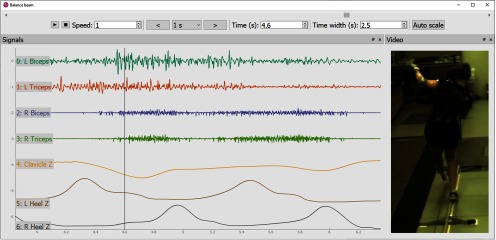
Using *neurotic* to explore balance beam walking EMG and kinematics. The vertical line corresponds to the time of the video frame shown where the participant lifts her left arm and extends it at the elbow as she regains balance while walking along a narrow beam (1.8 cm wide, 3.66 m long). Time-synched EMG signals show that the left triceps is activated before this event (second trace), consistent with extending the elbow, and then is followed by activation of the biceps (first trace), consistent with braking elbow extension. The *z*-coordinates (elevation) of the markers at the center of the clavicles and on each heel are also plotted. At the time of the illustrated video frame, the dip in clavicle elevation (fifth trace) is associated with the lowering of the participant’s right side of the torso as part of the balancing response. The left heel marker (sixth trace) shows that the left leg was nearing the ground just before being placed on the beam. The right heel marker (seventh trace) shows a slow rise as the participant began to roll forward on the ball of her right foot.

*neurotic* has broad applications because it uses the Neo Python library for reading a wide variety of file formats. For example, Figure 5 shows human EMG and kinematic data, stored as a binary MATLAB MAT-file, as a person maintains her balance while walking across a narrow beam. Left arm muscle activation timing corresponds to the moments that the participant could be seen correcting for a momentary balance loss. Similarly, a decrease in right shoulder elevation is seen simultaneously in both the video and a digitized record of a body marker tracking shoulder position in 3-D space.

## Discussion

Using *neurotic*, behavioral and neural responses to stimuli can be identified and annotated rapidly and subjected to initial signal processing. Changes in neural activity during swallowing on unloaded and loaded food stimuli in *Aplysia* could be readily observed (Fig. 4), and relationships between human movement and EMG were deducible (Fig. 5). In addition, dynamical model data could be readily incorporated and compared with empirical physiological data.

Open source projects like this thrive on contributions from volunteer developers. Because *neurotic* uses the Neo package for reading electrophysiology files, members of the NeuralEnsemble community (http://neuralensemble.org) can readily contribute to its further development. In general, open source projects depend on an enthusiastic community of developers, and if *neurotic* attracts such a community, it will continue to develop. The source code is stored on GitHub, which provides valuable collaboration tools such as issue tracking, pull request management, conversations, and task delegation. The open source model allows anyone to suggest improvements, submit bug fixes, or contribute new features; the community of maintainers seeks consensus on whether to accept contributions or request alterations; finally, changes are accepted only after passing a review process and automated testing. The complete history of the code can be found on GitHub, and prior releases of the software will remain available on distribution platforms like PyPI and Anaconda Cloud, so older versions of the software should always remain accessible.

There are trade-offs scientists should consider when choosing to use either free, open source software for their research or proprietary, closed source software. Commercial solutions that provide video synchronization capabilities similar to *neurotic* are few, and those proprietary alternatives may require expensive investments in software licenses and specific hardware. Maintaining proprietary systems depends on the continued financial viability of the company that makes them; old versions may stop working, and new hardware may be needed to upgrade software. For open source programs, maintainability again depends on the community. In some important ways, open source is superior for reproducibility and transparency, because the code is available for inspection, users suggest and often implement improvements to the code, and bugs are usually found and fixed quickly. Although proprietary systems often provide expensive “plug-ins” that can be added to the original software, open source projects excel at extensibility, because users can readily add functionality, or even specialize the program further for their own applications. The major investment for an open source project is the time that needs to be devoted to the project, whereas the major investment associated with proprietary software and hardware solutions is the money that needs to be invested into the hardware and software.

There are exciting possibilities for future development of *neurotic*. More signal processing options could be provided, such as customizable or even arbitrary multistage analysis procedures. Sound could be added to video playback. More advanced spike sorting techniques could be integrated [e.g., using results from tridesclous (tridesclous.readthedocs.io) or NeuroChaT (Islam et al., 2019)]. The pyqtgraph GUI library has many capabilities that could expand *neurotic’s* interactive and annotation features. Performance could be improved by incorporating GPU acceleration using the VisPy library (http://www.vispy.org). Finally, thanks to the Neo community, the list of compatible file formats is continuously growing.

## Synthesis

Reviewing Editor: Mark Laubach, American University Decisions are customarily a result of the Reviewing Editor and the peer reviewers coming together and discussing their recommendations until a consensus is reached. When revisions are invited, a fact-based synthesis statement explaining their decision and outlining what is needed to prepare a revision will be listed below. The following reviewer(s) agreed to reveal their identity: Andrew Davison.

Reviewer 1 wrote:

"My only concern is on how Quality control would be maintained in an open program like this one where users can modify/add functionalities. I do not understand how quality control is maintained in programs that are open in this way. I assume that is a resolved issue in the computer community, but perhaps, for idiots like me, the authors might mention how it will be done...will there be a moderator or something, or do updates/additions occur is such a way the all versions are continually available, etc.

With respect to fine editing, the figures were perfect on my screen and I was unable to find any typos or grammatical errors. I think the first time I have ever written that phrase.”

Reviewer 2 wrote:

"This is an excellent example of open science, in that (i) the software itself is open source, and builds on many existing open source software tools to produce results that would otherwise require two orders of magnitude more effort and resources; (ii) the software has features that explicitly promote sharing of analysis methods, and the reuse of existing datasets from public databases.

Installation was straightforward, and the user interface fairly intuitive, although some video-based documentation would be helpful for novice users.

I have a few concerns about the manuscript, primarily about how it situates the work in a broader context.

(1) In the Introduction, there is the statement “there are few free and open-source software tools with this capability”. Which tools specifically do have this capability? How does *neurotic* compare to them? Are there any MATLAB-based packages that are equivalent to *neurotic*?

(2) This sentence is seemingly in contradiction with the following one, which states “there are many open source neuroscience tools and libraries”. Presumably, the “many” refers to libraries/tools that can perform a subset of the capabilities of *neurotic*? This should be clarified, and examples given of the tools/libraries you are referring to.

(3) The Discussion is very short, and is missing in particular a discussion of the advantages and disadvantages of *neurotic* with respect to similar tools, e.g. Plexon Cineplex, Blackrock NeuroMotive. What considerations should a behavioural neuroscientist bear in mind when choosing such software? Aspects to be discussed should include support, maintainability, reproducibility, extensibility.”

Review Synthesis (Essential Revisions):

1 - Please address the three points raised by R#2 in your rebuttal and also revise the manuscript to address each point. Please be sure to denote exactly where the changes were made in the revised manuscript.

2 - Please also address how quality control can be maintained given the open nature of the project, as raised by R#1. Again, please be sure to denote where changes were made in the manuscript to address this issue.
